# Moral processing deficit in behavioral variant frontotemporal dementia is associated with facial emotion recognition and brain changes in default mode and salience network areas

**DOI:** 10.1002/brb3.843

**Published:** 2017-11-29

**Authors:** Jan Van den Stock, Daphne Stam, François‐Laurent De Winter, Dante Mantini, Benedikt Szmrecsanyi, Koen Van Laere, Rik Vandenberghe, Mathieu Vandenbulcke

**Affiliations:** ^1^ Laboratory for Translational Neuropsychiatry Department of Neurosciences KU Leuven Leuven Belgium; ^2^ Department of Old Age Psychiatry University Psychiatry Center Leuven Belgium; ^3^ Research Center for Movement Control and Neuroplasticity KU Leuven Leuven Belgium; ^4^ Department of Health Sciences and Technology ETH Zurich Zurich Switzerland; ^5^ Department of Experimental Psychology University of Oxford Oxford UK; ^6^ Quantitative Lexicology and Variational Linguistics KU Leuven Leuven Belgium; ^7^ Nuclear Medicine and Molecular Imaging Department of Imaging and Pathology KU Leuven Leuven Belgium; ^8^ Laboratory for Cognitive Neurology Department of Neurosciences KU Leuven Leuven Belgium; ^9^ Department of Neurology University Hospitals Leuven Leuven Belgium

**Keywords:** emotion processing, fractional amplitude of low‐frequency fluctuations, frontotemporal dementia, insula, moral processing, social cognition

## Abstract

**Introduction:**

Behavioral variant frontotemporal dementia (bvFTD) is associated with abnormal emotion recognition and moral processing.

**Methods:**

We assessed emotion detection, discrimination, matching, selection, and categorization as well as judgments of nonmoral, moral impersonal, moral personal low‐ and high‐conflict scenarios.

**Results:**

bvFTD patients gave more utilitarian responses on low‐conflict personal moral dilemmas. There was a significant correlation between a facial emotion processing measure derived through principal component analysis and utilitarian responses on low‐conflict personal scenarios in the bvFTD group (controlling for MMSE‐score and syntactic abilities). Voxel‐based morphometric multiple regression analysis in the bvFTD group revealed a significant association between the proportion of utilitarian responses on personal low‐conflict dilemmas and gray matter volume in ventromedial prefrontal areas (*p*_height_ < .0001). In addition, there was a correlation between utilitarian responses on low‐conflict personal scenarios in the bvFTD group and resting‐state fractional Amplitude of Low Frequency Fluctuations (fALFF) in the anterior insula (*p*_height_ < .005).

**Conclusions:**

The results underscore the importance of emotions in moral cognition and suggest a common basis for deficits in both abilities, possibly related to reduced experience of emotional sensations. At the neural level abnormal moral cognition in bvFTD is related to structural integrity of the medial prefrontal cortex and functional characteristics of the anterior insula. The present findings provide a common basis for emotion recognition and moral reasoning and link them with areas in the default mode and salience network.

## 1 INTRODUCTION

Behavioral variant frontotemporal dementia (bvFTD) is a neurodegenerative disorder typically associated with changes in personality and behavior, accompanied by fronto‐temporal and subcortical atrophy. Deficits in emotion recognition in bvFTD have been reported consistently and in multiple modalities like facial (Rosen et al., 2002), bodily (Van den Stock, De Winter, et al., 2015) and musical expressions (Omar et al., 2011). This has led to the hypothesis that there is a common basis for these multimodal socio‐cognitive deficits. Clinically, one of the most striking symptoms of bvFTD patients is early loss of appropriate emotional reactions to salient events. For instance, bvFTD patients may react undisturbed to incidents which normally would trigger significant emotional reactions, e.g., losing their job (Miller, 2014). This example also illustrates that the deficit cannot be confined to “affective empathy” (Rankin, Kramer, & Miller, 2005), as it also pertains for events outside a social context. A deficit in emotional experience, i.e., feelings (Damasio & Carvalho, 2013) may indeed constitute the common underlying basis of multimodal emotion recognition deficits. Furthermore, should this indeed be the case, it can be hypothesized that it has a similar influence on other higher order socio‐cognitive deficits like moral reasoning abnormalities (Baez, Kanske, et al., 2016; Baez, Morales, et al., 2016; Chiong et al., 2013; Mendez, 2006, 2009; Mendez, Anderson, & Shapira, 2005; Mendez & Shapira, 2009).

Assessment of moral reasoning typically consists of presenting subjects with moral dilemmas and one or more action alternatives. A classical example is the trolley dilemma (Foot, 1967). It states that a runaway trolley is rapidly approaching five workers on the railway track. These five men can be saved, by pulling a lever, which will divert the trolley toward another track, where it will kill one other railway worker. The question for the subject is: “would you pull the lever so the trolley will kill one person instead of 5?”. This type of dilemma has been used to assess, for instance, utilitarian moral reasoning and how it is influenced by the degree of conflict between the utilitarian benefit and the emotional aversion that is associated with the proposed action (high vs. low‐conflict dilemmas).

Contemporary accounts of moral processing increasingly put emphasis on the emotional underpinnings of moral reasoning, in addition to the rational factors. A current dominant view on moral processing makes a distinction between “personal” (and putatively highly emotional) and “impersonal” (and putatively less emotional) moral thinking (Greene, Sommerville, Nystrom, Darley, & Cohen, 2001). A moral violation is personal if it causes serious bodily harm to a particular person, harm which does not result from the deflection of an existing threat onto a different party (Greene & Haidt, 2002), e.g., pushing a large person onto the tracks to stop a runaway trolley from killing five other people on the rail. A moral violation that does not fulfill these criteria is considered impersonal, e.g., hitting a switch that will divert the trolley to a different set of tracks where it will kill only one person instead of five. There is much debate on how emotional and cognitive processes interact during moral reasoning. Impersonal moral dilemmas are thought to be driven by conscious cognitive processes (prefrontal regions) and require a utilitarian calculation of how to maximize welfare while “personal” moral dilemmas are likely driven by automatic emotional responses (limbic regions) (Greene, Nystrom, Engell, Darley, & Cohen, 2004; Moll, De Oliveira‐Souza, & Zahn, 2008) and psychologically reflect socio‐emotional processes. A study by Koenigs et al. (2007) provided support for the hypothesis that emotions play a crucial role in the generation of moral judgments. They observed that patients with damage to the ventromedial prefrontal cortex (vmPFC) showed an increased “utilitarian” pattern of judgments on personal but not impersonal moral dilemmas. Furthermore, the deficit was specific for so‐called “high‐conflict” personal dilemmas. High‐conflict personal moral dilemmas oppose aggregate welfare to inflicting harm on others by means of a highly (emotionally) aversive action, e.g., smothering one's baby to save a group of people. In “low‐conflict” personal dilemmas, the proposed utilitarian action is less emotional, e.g., ignoring the plea for help from a bleeding man at the side of the road for the sake of preserving the leather upholstery of one's car.

Interestingly, bvFTD is also associated with increased utilitarian judgments and autonomic reactivity on personal, but not on impersonal moral dilemmas (Chiong et al., 2013; Fong et al., 2016; Mendez & Shapira, 2009), although it is not known whether the deficit is related to high versus low‐conflict personal dilemmas. Considering the significant emotional load in personal moral dilemmas, combined with the emotional recognition deficits in bvFTD (Kumfor & Piguet, 2012), this specific abnormality in judgments of personal moral dilemmas may link with a deficit in emotional recognition. However, no empirical association between emotion recognition and moral reasoning has been reported so far. In the present study, our aim was to fill these gaps in the literature and increase the knowledge of moral deficits in bvFTD. For this purpose, we manipulated the level of conflict in personal moral dilemmas (high‐ vs. low‐conflict). This allows a critical test of two conflicting hypotheses relating to the nature of emotional‐moral deficits in bvFTD. The first one we term the “threshold”‐hypothesis: if emotional deficits in bvFTD show a progression from subtle to manifest emotional cues and endorsement of a utilitarian action requires overcoming a critical level of emotional aversion against inflicting direct harm to another person, then a deficit on low‐conflict personal dilemmas would precede a deficit on high‐conflict personal dilemmas (which require a more severe blunting to emotional cues). Support for this hypothesis comes from studies showing that bvFTD impairs recognition of low‐ but not high‐intense emotional expressions (Jastorff et al., 2016; Kumfor et al., 2011) On the other hand, if bvFTD results in a reduced recognition of emotion cues regardless of intensity (i.e., from subtle to extreme), then a deficit on both low‐ and high‐conflict moral dilemmas would be expected (“overall”‐hypothesis). Support for the “overall”‐hypothesis comes from a study documenting impaired recognition of caricature (i.e., exaggerated) facial expressions (Kumfor, Irish, Hodges, & Piguet, 2013). To further document the nature of moral deficits in bvFTD, particularly a possible involvement of emotional processes in moral judgments, the present study investigates for the first time associations between measures of emotion recognition and moral reasoning.

The second aim was to investigate the associated neuro‐anatomy of personal moral processing deficits in bvFTD, which has not been explored hitherto. There is evidence from other moral reasoning paradigms that moral processing abnormalities in bvFTD are primarily associated with regions in the salience and default mode network (anterior cingulate, ventromedial prefrontal and posterior cingulate cortex, temporo‐parietal junction) (Baez, Kanske, et al., 2016; Baez, Morales, et al., 2016; Chiong et al., 2013). Analogous to findings in vmPFC patients, we hypothesize a critical involvement of the vmPFC (Koenigs et al., 2007). The novel aspect that the present study adds to the field is the functional brain characterization of moral processing deficits in bvFTD by means of fractional Amplitude of Low‐Frequency Fluctuations (fALFF) (Zou et al., 2008). fALFF is a measure of regional brain activation over time and across the entire brain and has been proven a valuable biomarker in neurodegenerative disorders (Han et al., 2012; Mascali et al., 2015).

## 2 METHODS

### 2.1 Participants

Thirteen patients diagnosed with probable bvFTD and 19 healthy controls took part in the study. Patients were recruited from the memory clinic and the Old Age Psychiatry Department of University Hospitals Leuven (Leuven, Belgium) (*N* = 8) as well as from the Neurology Department at the regional Onze‐Lieve‐Vrouw Ziekenhuis Aalst‐Asse‐Ninove (Aalst, Belgium) (*N* = 5). Diagnoses were made by experienced neurologists or old age psychiatrists after clinical assessment, collateral history, cognitive neuropsychological testing, and suggestive patterns of atrophy on structural MRI. In 11 patients, diagnosis was also based on a typical pattern of hypo‐metabolism on a ^[18F]^Fluorodeoxyglucose PET scan. All patients fulfilled the criteria for “Probable bvFTD” (Rascovsky et al., 2011). Patients initially presented with changes in behavior and personality displaying disinhibition, apathy and/or perseverative/compulsive behavior. At inclusion, mean symptom duration assessed by hetero‐anamnesis equaled 2.11 years (*SD* = 1.04). Patients were included after clinical judgment deemed them able to successfully undergo an experimental scanning session. Note that an additional six patients agreed to participate, but no experimental scanning data could be acquired due to a lack of cooperation and/or agitation. Genotyping for known mutations was performed in six patients (GRN & C9orf72 = 3; GRN = 2; GRN & MAPT = 1). All results of the genetic analyses were negative. All participants also took part in our previous studies on bvFTD (De Winter, Timmers, et al., 2016; De Winter, Van den Stock, et al., 2016; Jastorff et al., 2016; Van den Stock, De Winter, et al., 2015).

Healthy control subjects were recruited through a database of elderly volunteers as well as through advertisements in a local newspaper. Exclusion criteria included present or past neurological or psychiatric disorders including substance abuse as well as significant systemic comorbidities or use of medication susceptible to affect the central nervous system.

Cognitive neuropsychological testing was conducted in all participants and included evaluation of global cognitive ability (Mini Mental State Examination) (Folstein, Folstein, & McHugh, 1975), verbal memory (Rey's Auditory Verbal Learning Test) (Rey, 1958), categorical verbal fluency (animal verbal fluency) (Lezak, Howieson, & Loring, 2004), abstract reasoning ability (Raven's colored progressive matrices A and B) (Raven, 1995), visual divided attention and task shifting (Trail Making Test A and B) (Reitan, 1958), low‐level aspects of visual perception [Birmingham Object Recognition Battery (BORB) (Riddoch & Humphreys, 1993): length, size and orientation matching], confrontation naming (Boston Naming Test) (Kaplan, Goodglass, & Weintraub, 1983), and language comprehension (comprehension subsection of Aachen Aphasia Test) (Weniger, Willmes, Huber, & Poeck, 1981).

The study was conducted according to the Declaration of Helsinki and approved by the ethical committee of University Hospitals Leuven, Belgium. All subjects gave written informed consent. All subjects had normal or corrected‐to‐normal visual acuity. All participants were right‐handed as assessed through the Edinburgh Handedness Inventory. Demographic and clinical data are presented in Table 1.

**Table 1:** Demographic and clinical data

	bvFTD (*N* = 13)	Ctrl (*N* = 19)	*p*
Gender (♂/♀)	9/4	11/8	.780
Age	66.6 (7.22)	66.5 (6.28)	.978
Disease duration in years	2.10 (1.04)	n/a	
MMSE (/30)^b^	26.6 (1.57)	29.3 (0.650)	.001
RAVLT^b^			
A1–A5 (/75)^b^	29.5 (9.43)	50.9 (7.52)	.001
% Delayed recall (/100)^b^	57.6 (34.7)	80.4 (17.68)	.044
Recognition (/15)^b^	7.20 (6.87)	14.0 (1.37)	.004
AVF^b^	14.9 (6.11)	22.5 (5.78)	.001
RCPMT (/24)^b^	16.9 (4.17)	20.7 (2.85)	.005
TMT^b^			
A^b^	68.0 (50.4)	32.6 (9.69)	.027
B	186 (149)^a^	90.5 (4.51)	.061
BORB			
Length match task (% correct)	86.7 (7.70)	90.2 (4.51)	.157
Size match task (% correct)	86.7 (6.09)	88.9 (6.29)	.316
Orientation match task (% correct)	80.8 (10.2)	86.1 (6.01)	.074
BNT (/60)^b^	39.7 (13.2)	54.3 (3.00)	.002
AAT^b^			
Comprehension (/120)^b^	94.1 (12.9)	109 (5.34)	.001

AAT, Aachen Aphasia Test; AVF, Animal Verbal Fluency; BNT, Boston Naming Test; BORB, Birmingham Object Recognition Battery; RCPM, Raven's Coloured Progressive Matrices; MMSE, Mini‐mental state examination; n/a, not applicable; TMT, Trail Making Test. RAVLT, Rey Auditory Verbal Learning Test; A1–A5, sum of trials A1 to A5; % Delayed recall, Delayed recall/(maximum (A1–A5)) * 100; Recognition, correct hits – false hits.

a *N* = 11.

b Significant group differences.

### 2.2 Behavioral assessment

#### 2.2.1 Judgment of moral dilemmas

Subjects were presented with 50 verbal descriptions of hypothetical scenarios (see: http://www.nature.com/nature/journal/v446/n7138/extref/nature05631-s1.pdf). All the scenarios have a very similar grammatical structure and each scenario ends with the question whether the subject would perform a hypothetical action in the respective scenario, which subjects were instructed to answer. The scenarios were categorized into three groups: non‐moral (involving practical dilemmas; *n* = 18), impersonal moral (involving impersonal weighting of harms and benefits; *n* = 11), and personal moral (involving utilitarian infringements of personal rights; *n* = 21) (Greene et al., 2001). The latter category was subdivided into two subcategories, based on a validation study in normal subjects (Koenigs et al., 2007). The subdivision was related to the magnitude of the conflict between the utilitarian benefit and the emotional aversion that is associated with the proposed action: high (*n* = 13) versus low‐conflict (*n* = 8) dilemmas. The scenarios were translated into Dutch by a registered translation company and the procedure was further similar to the one described in (Koenigs et al., 2007).

In what follows we provide examples of each of the four moral conditions:

☐  Nonmoral dilemma: “You are bringing home a number of plants from a store that is about two miles from your home. The trunk of your car, which you've lined with plastic to catch the mud from the plants, will hold most of the plants you've purchased. You could bring all the plants home in one trip, but this would require putting some of the plants in the back seat as well as in the trunk. By putting some of the plants in the back seat you will ruin your fine leather upholstery which would cost thousands of dollars to replace. Would you make two trips home in order to avoid ruining the upholstery of your car?”
☐  Impersonal moral dilemma: “You work for the Bureau of Health, a government agency. You are deciding whether or not your agency should encourage the use of a certain recently developed vaccine. The vast majority of people who take the vaccine develop an immunity to a certain deadly disease, but a very small number of people who take the vaccine will actually get the disease that the vaccine is designed to prevent. All the available evidence, which is very strong, suggests that the chances of getting the disease due to lack of vaccination are much higher than the chances of getting the disease by taking the vaccine. Would you direct your agency to encourage the use of this vaccine in order to promote national health?”
☐  Personal low‐conflict moral dilemma: “You are driving along a country road when you hear a plea for help coming from some roadside bushes. You pull over and encounter a man whose legs are covered with blood. The man explains that he has had an accident while hiking and asks you to take him to a nearby hospital. Your initial inclination is to help this man, who will probably lose his leg if he does not get to the hospital soon. However, if you give this man a lift, his blood will ruin the leather upholstery of your car. Would you leave this man by the side of the road in order to preserve your leather upholstery?”
☐  Personal high‐conflict moral dilemma: “Enemy soldiers have taken over your village. They have orders to kill all remaining civilians. You and some of your townspeople have sought refuge in the cellar of a large house. Outside you hear the voices of soldiers who have come to search the house for valuables. Your baby begins to cry loudly. You cover his mouth to block the sound. If you remove your hand from his mouth his crying will summon the attention of the soldiers who will kill you, your child, and the others hiding out in the cellar. To save yourself and the others you must smother your child to death. Would you smother your child in order to save yourself and the other townspeople?”

The stimuli were presented using presentation ^®^ software.

#### 2.2.2 Emotion processing

A series of psychophysical experiments was conducted to assess emotion processing across category (eyes, faces, bodies), motion (static, dynamic), and task (detection, discrimination, matching, selection, categorization). Only a brief description of every experiment is given here, as all procedures have been described in detail elsewhere.

##### Adapted Reading the mind in the eyes test (RMET)

We adapted the RMET to a simultaneous 2‐alternative forced‐choice match‐to‐sample task. Patients are presented with a rectangular picture showing a pair of eyes on top and two pairs of eyes below. They were instructed to indicate which expression below best matched the expression on top. The stimuli were constructed based on the semantic relation of the originally associated accurate verbal labels (Baron‐Cohen, Wheelwright, Hill, Raste, & Plumb, 2001). The full list of composition of the 13 trials is presented in Table S1.

##### Facial emotion detection

This task consists of simultaneous presentation of a neutral and an emotional face (with varying intensities of emotion) with the same identity while subjects are instructed to indicate the emotional face (De Winter, Van den Stock, et al., 2016).

##### Facial emotion discrimination

This task is a subtest from the Florida Affect Battery (FAB) (Bowers, Blonder, & Heilman, 1999) and consists of simultaneous presentation of two facial expressions. The subject has to indicate whether both pictures express the same emotion.

##### Static facial emotion matching

Subjects are asked which of two emotional faces displayed at the bottom express the same emotion as a third face displayed on top of the screen. The three faces on display always had a different identity (de Gelder, Huis in ‘t Veld, & Van den Stock, 2015).

##### Static bodily emotion matching

The procedure here is similar to the static facial emotion matching experiment, but with stimuli of bodies instead of faces (de Gelder & Van den Stock, 2011).

##### Dynamic facial emotion matching

The procedure here is similar to the static facial emotion matching experiment, but with dynamic instead of static stimuli (Zhu et al., 2013).

##### Dynamic bodily emotion matching

The procedure here is similar to the dynamic facial emotion matching experiment, but with stimuli of bodies instead of faces (Van den Stock, De Winter, et al., 2015).

##### Facial emotion selection

Subjects are instructed to indicate which of five facial expressions matches a verbal label. This is a subtest from the FAB (Bowers et al., 1999).

##### Facial emotion categorization

Subjects are instructed to indicate which of five verbal labels matches a picture of a facial expression. This is a subtest from the FAB (Bowers et al., 1999).

Stimuli of all these tasks except for the RMET and those from the FAB were presented using presentation ^®^ software.

### 2.3 Brain imaging

All subjects were scanned on a single 3 Tesla Philips Achieva scanner using a 32‐channel head coil.

#### 2.3.1 Structural brain imaging

A high resolution anatomical scan (TR 9.6 ms, TE 4.6 ms, flip angle 8°, 182 slices, matrix size 256 × 256 and 0.98 × 0.98 × 1.20 mm^3^ voxel size) was acquired with coronal slice orientation.

#### 2.3.2 Functional brain imaging

A resting‐state scan was performed (TR: 1,700 ms; TE: 33 ms; matrix size: 64 × 64; FOV: 230 mm; flip angle: 90°; slice thickness: 4 mm; no gap; axial slices: 32), consisting of 250 functional volumes with a total duration of 7 min. During the scan all subjects were instructed to close their eyes and lie as still as possible, while not falling asleep or thinking of anything in particular.

### 2.4 Brain imaging analysis

Brain imaging data were analyzed using Brainvoyager 20.2, SPM12 (Wellcome Trust Centre for Neuroimaging, UCL, London, UK) running under MATLAB R2008a, NeuroElf v11_6401 (www.neuroelf.net) and in‐house developed processing routines.

#### 2.4.1 Structural

T1‐weighted structural images were reoriented to the ACPC plane and centered on the anterior commissure. The VBM8 toolbox (http://dbm.neuro.uni-jena.de/vbm/) was used for structural image analysis. Preprocessing included bias correction, segmentation, and normalization to MNI space within a unified model, including high‐dimensional DARTEL‐normalization. In order to compensate for the effect of normalization and preserve absolute tissue volumes the resulting normalized gray matter (GM) segmentations underwent Jacobian modulation. Modulated images were smoothed using a Gaussian kernel of 8 mm at FWHM.

#### 2.4.2 Functional

Preprocessing of the functional scans included slice time scan correction by means of cubic spline interpolation, 3D motion correction by means of trilinear/sinc interpolation and linear trend temporal filtering. We used an adaptation of Amplitude of Low Frequency Fluctuation as a measure of variability in resting‐state activation (Zang et al., 2007). In ALFF analyses, voxel time series are bandpass filtered (0.01–0.08 Hz) to remove the effects of very‐low‐frequency drift and high frequency noise. Subsequently, the time series are transformed to a frequency domain with a fast Fourier transform to obtain the power spectrum. The square root is then calculated at each frequency of the power spectrum to obtain the voxel‐wise averaged square root across 0.01–0.08 Hz. These values are then normalized by division by the global mean. Finally, the ratio is computed of the power of each frequency at the low‐frequency range (0.01–0.08 Hz) to that of the entire frequency range (0–0.25 Hz) which results in the fractional Amplitude of Low‐Frequence Fluctuation (fALFF) (Zou et al., 2008).

## 3 RESULTS

### 3.1 bvFTD atrophy

**Figure 1:**
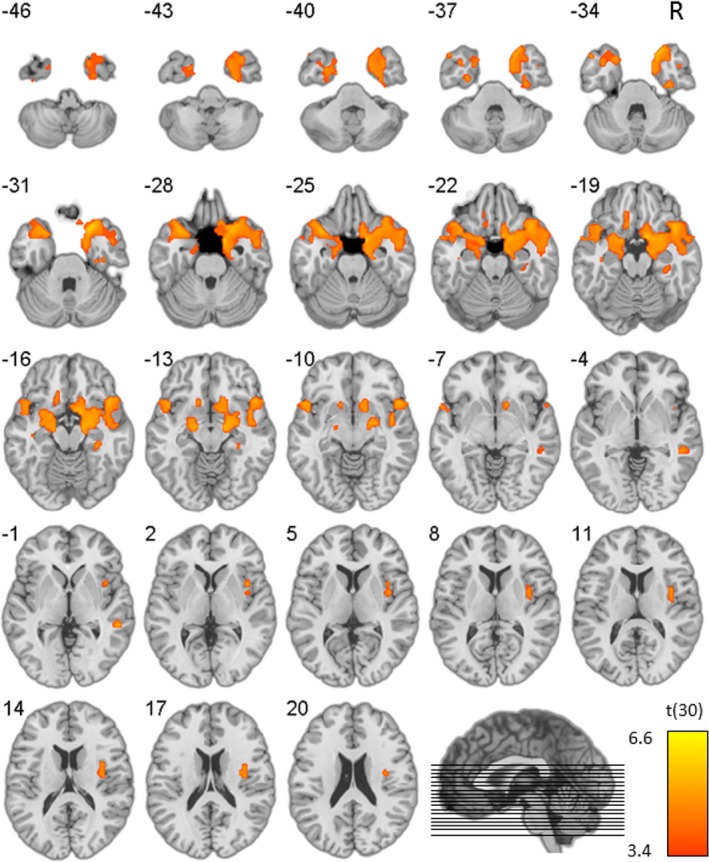
Statistical map displaying group difference in gray matter (GM) volume (Ctrl > bvFTD), overlaid on a normal template. *Z*‐coordinates in MNI space, *p*_height_ < .001

GM images were entered in a general linear model and a two sample *t*‐test was performed for a whole‐brain group comparison (*p*_height_ < .001, clustersize > 100 voxels). The results are displayed in Figure 1 and reveal reduced gray matter volume in the bvFTD group in anterior temporal, subcortical, and to a lesser extent frontal regions.

Visual inspection of the individual scans by an experienced clinician (MV) revealed that the sample was composed of patients with primarily temporal (*N* = 7), primarily frontal (*N* = 3) and fronto‐temporal (*N* = 3) atrophy.

### 3.2 Behavioral analysis

Normality testing was performed on all variables using a Shapiro–Wilk test (alpha set at .05). On normally distributed data two‐sample *t*‐tests were performed to investigate group effects, preceded by Levene's tests to test homoscedasticity. If the null hypothesis of equal variances was rejected, Welch's *t*‐test was used (an adaptation of Student's *t*‐test which accounts for unequal variances). In case a normal distribution could not be assumed on all variables of an Experiment, Mann–Whitney *U* tests were performed instead.

#### 3.2.1 Moral judgments

For the nonmoral judgments, we counted for every subject the number of “yes” responses. The moral judgment responses (i.e., yes or no) of every subject were coded as a function of utilitaristic or not. As a first control test, we investigated whether the controls differed from the patients on the nonmoral condition by means of Mann–Whitney *U* Test. This revealed no significant group difference (*U* = 103, *p* = .448). A significant group difference on the nonmoral condition would question the validity of the results on the moral conditions. Subsequently, we investigated whether the patients gave more utilitarian responses on the two moral conditions. This revealed no significant difference for the impersonal (*U* = 124, *p* = .999) and personal (*U* = 115, *p* = .734) moral conditions. Subsequently, we investigated increased utilitarian responses in the bvFTD group on the personal moral dilemmas as a function of conflict level. This revealed a significant difference on the low‐conflict (*U* = 70, *p* = .021, one‐tailed, uncorrected), but not on the high‐conflict dilemmas (*U* = 136, *p* = .650) condition. Furthermore, to determine if the linguistic complexity of the original verbal descriptions in English is unduly variable, we used the Coh‐Metrix tool at http://cohmetrix.com/ to calculate a battery of 106 customary linguistic complexity indices per scenario. The indices cover grammatical, lexical, psycholinguistic, and semantic complexity, as well as readability. We performed a series of Mann–Withney *U* tests on these 106 variables, comparing the low‐ with the high‐conflict condition. To control for type 1 errors when performing 106 comparisons, we set the alpha‐level at .001. This revealed a significant difference on only two variables. The first one (LSASSpd) is one of eight measures of semantic overlap between sentences. It reflects the standard deviation of Latent Semantic Analysis (Landauer, McNamara, Dennis, & Kintsch, 2007) cosine of all sentence pairs within paragraphs. The second one (WRDFRQc) reflects the average word frequency for content words. We thus conclude that the linguistic complexity of the descriptions is largely equal, and reasonably assume that the same holds for the Dutch translations. Both Dutch and English are part of the Germanic branch of the Indo‐European language family. The Dutch verb system has similar tenses to English and is similarly uninflected. Furthermore, both languages follow the same basic Subject‐Verb‐Object form and use definite and indefinite articles in much the same way. In addition, all moral dilemmas are composed of short sentences, with a minimum of grammatical complexity.

**Figure 2:**
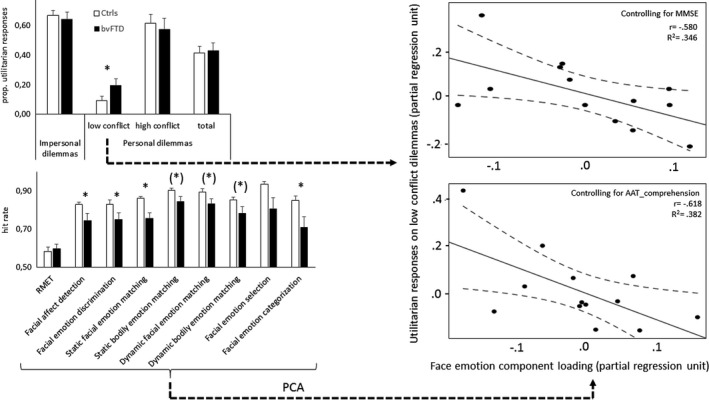
Behavioral results and schematic analysis procedure. The bar charts on the left display performance on the moral judgment (top) and emotion processing (bottom) experiments. The scatterplots on the right display the partial correlation between utilitarian responses on low‐conflict dilemmas (*Y*‐axis) and loading on the face emotion component derived from the emotion processing experiments (*X*‐axis), controlling for MMSE‐score (top scatter plot) and AAT_comprehension‐score (bottom). The scatterplots include the linear fitted line (full line) and 95% confidence interval of the mean. RMET, adapted Reading the Mind in the Eyes Test; PCA, Principal Component Analysis; *p < .05; (*)*p* < .059

The results of the moral judgments as a function of group, personal affiliation (impersonal and personal) and conflict level are displayed in Figure 2.

#### 3.2.2 Emotion processing

For every experiment, we first computed the mean response time and standard deviation of the response times for every subject. Subsequently, we identified the trials in which the RT exceeded the mean (subject‐specific) RT by at least 3 (subject‐specific) standard‐deviations. These trials were then excluded from further analysis. Significant group differences were observed on facial emotion detection (*t*(14.637) = 2.216, *p* = .043), facial emotion discrimination (*t*(30) = 2.126, *p* = .042), static facial emotion matching (*t*(14.463) = 3.542, *p* = .003), and facial emotion categorization (*U* = 71, *p* = .045). There was a trend for group differences in static (*U* = 70, *p* = .059) and dynamic (*U* = 74.5, *p* = .059) body emotion matching and for dynamic face emotion matching (*U* = 73.5, *p* = .054). There were no clear group differences on RMET (*U* = 140.5, *p* = .520) and face emotion selection (*U* = 85, *p* = .147).

#### 3.2.3 Moral‐emotion processing correlation

As a first step in investigating an association between abnormal moral processing and emotion processing capacities in bvFTD, we performed a variable reduction of the emotion processing experiments. We conducted a principal component analysis (PCA) on the scores of all emotion processing experiments. The inclusion of all experiments instead of only the ones showing a group difference, has the advantage that it may capture more nuanced aspects of emotion processing, as it is based on a conceptually more heterogeneous set of variables. Furthermore, deficits in bvFTD have been reported on all these measures (Gregory et al., 2002; Kumfor, Hazelton, De Winter, Cleret de Langavant, & Van den Stock, 2017; Rosen et al., 2002; Van den Stock, De Winter, et al., 2015).

The number of withheld components of the PCA was a priori restricted to the ones with an eigenvalue >1 and a direct oblimin rotation procedure with Delta = 0 was used. This resulted in an oblique 2‐component model (for details, see Table 2), explaining 78% of the total variance. The correlation between the components equalled ‐.47. The pattern matrix (Table 2) reveals that static facial emotion recognition, facial emotion selection, and facial emotion discrimination show the highest loadings on the first component, while the Reading the Mind in the Eyes score loads highest on the second component (all loadings > .9). In line with the stimulus characteristics, we descriptively label these the face emotion and eye emotion components respectively. Subsequently, we performed a partial correlation analysis between the component loadings and the low‐conflict personal moral dilemma score, controlling for global cognitive capacity (MMSE‐score). This revealed a significant negative correlation with the face emotion component (*r*_(low conflict score‐face emotion component, MMSE)_ = −.588, *p* = .044), but not with the eye emotion component (*r*_(low conflict score‐eye emotion component, MMSE)_ = .364, *p* = .245). This indicates that more utilitarian responses are associated with lower face emotion processing scores, accounting for general cognitive decline.

**Table 2:** Component loadings and communalities based on a principal components analysis with oblimin rotation for nine emotion processing variables in bvFTD

		Component		
		1	2	Comm.
	Eigenvalue	5.59	1.38	
Motion				
Static	Reading the mind in the eyes test	0.25	0.93	0.74
Static	Facial affect detection	0.51	−0.57	0.82
Static	Facial emotion discrimination	0.91		0.82
Static	Facial emotion matching	0.58	−0.47	0.79
Static	Facial emotion selection	0.95		0.83
Static	Facial emotion categorization	0.97		0.88
Static	Bodily emotion matching	0.34	−0.67	0.75
Dynamic	Facial emotion matching	0.64		0.53
Dynamic	Bodily emotion matching	0.25	−0.77	0.82

Component loadings < 0.2 are suppressed. Comm., communalities.

In addition to controlling for general global cognitive capacity as measured the MMSE‐score, we also performed a partial correlation analysis in which we controlled for semantic capacity, i.e., language comprehension, as measured in the subtest “comprehension” of the Aachen Aphasia Test (AAT_comprehension). This also revealed a significant correlation with the face emotion component (*r*_(low conflict score‐face emotion component, AAT_comprehension)_ = −.618, *p* = .032), but not with the eye emotion component (*r*_(low conflict score‐eye emotion component, AAT_comprehension)_ = .267, *p* = .401). The results are displayed in Figure 2.

### 3.3 Brain‐behavior correlations

#### 3.3.1 Structural

**Figure 3:**
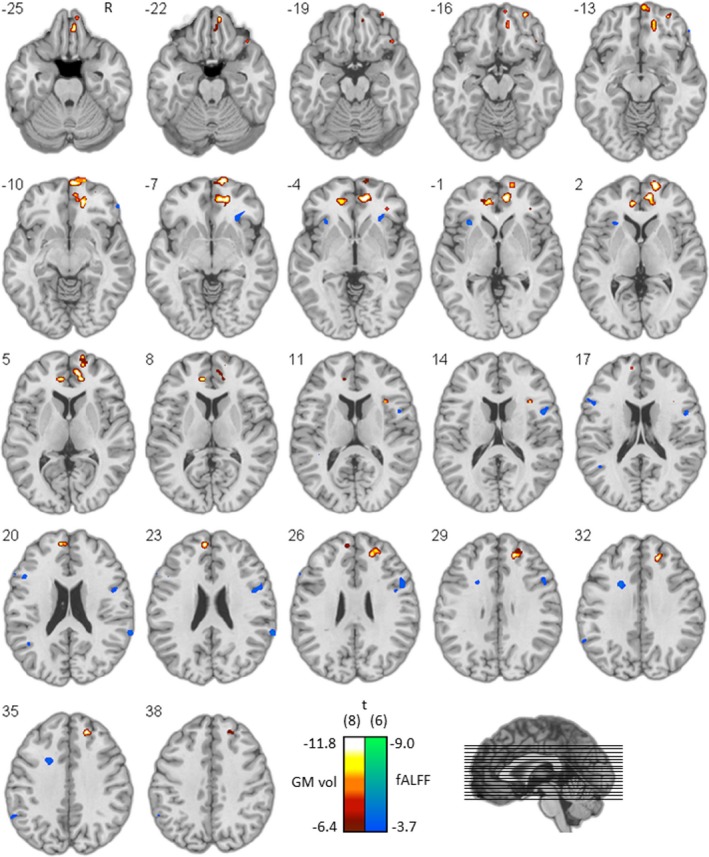
Brain‐behavior results. Statistical maps displaying association between proportion utilitaristic responses on the one hand and gray matter volume (red to white color coding) and resting‐state activity fluctuations (blue to green color coding) on the other hand, overlaid on a normal template. Coordinates refer to MNI‐space

The GM maps were submitted to a multiple regression analysis in which the score on low‐conflict moral dilemmas (i.e., the proportion utilitarian responses) was entered as covariate in order to investigate correlations between performance and voxel‐wise GM volume (*p*_height_ < .0001, minimal cluster size = 50 voxels). Age, gender and total intracranial volume (TIV) were entered as covariates of non‐interest. Significant results were primarily located in bilateral ventromedial prefrontal cortex and frontal operculum/anterior insula (see Figure 3 and Table S2).

#### 3.3.2 Functional

The fALFF maps were submitted to a multiple regression analysis in which the score on low‐conflict moral dilemmas was entered as covariate in order to investigate correlations between performance and voxel‐wise fALFF (*p*_height_ < .005, minimal cluster size = 10 voxels). Age and gender were entered as covariates of noninterest. Significant results were primarily located in the right frontal operculum/anterior insula (see Figure 3 and Table S2).

## 4 DISCUSSION

The present results reveal that increased utilitaristic moral behavior is associated with decreased face emotion recognition in bvFTD. The control analyses we performed did not provide any evidence that global cognitive decline or semantic abilities could explain this link, as we statistically controlled for these confounds. The correlation between moral deficits and face emotion recognition is in line with current views on moral cognition, which emphasize the importance of emotional underpinnings in moral behavior (Damasio, 1994; Haidt, 2001). Here, we report that deterioration of moral reasoning is associated with abilities in facial emotion recognition. We hypothesize that a diminished subjective experience of emotional sensations links emotion recognition and moral cognition impairment. Notably, reduced experience of physical sensations like pain and temperature have also been reported in bvFTD (Fletcher et al., 2015). Reduced experience of emotional sensations is a key clinical manifestation of bvFTD, which extends empathic deficits (Rascovsky et al., 2011). Both emotion recognition and moral cognition have been associated with empathic abilities in the normal population (Bzdok et al., 2012; Van den Stock, Hortensius, Sinke, Goebel, & de Gelder, 2015) as well as in bvFTD (Baez et al., 2014; Rankin et al., 2005). As the present main finding is correlational in nature, no claims can be made regarding the causal and consequential socio‐cognitive deficits in bvFTD and – by extension – in other syndromes associated with deficits in emotion recognition, moral cognition, and feelings.

The moral deficit in bvFTD was specific for low‐conflict and not high‐conflict personal dilemmas. It is unlikely that this pattern is explained by cognitive deficits, such as impaired language or executive functions, as these have a similar influence in all conditions. A similar argument can be made regarding the confounding influence of mentalizing deficits, as these also equally apply to the high‐conflict condition. It has been proposed that an emotional response (preventing direct harm to others) has to be overcome in order to enable utilitaristic behavior (Greene et al., 2001, 2004), and that this threshold is increased for high‐conflict compared to low‐conflict personal dilemmas. It is possible, then, that the emotional response triggered by the high‐conflict dilemmas was too strong to be overcome in our bvFTD sample. At the same time, subjects were more easily inclined towards utilitaristic behavior in low‐conflict dilemmas, where the emotional response was less salient. The present results thus support the “threshold”‐hypothesis for moral‐emotion deficits in bvFTD stating that recognition deficits of subtle emotion cues precede those of more intense emotion signals (Jastorff et al., 2016; Kumfor et al., 2011).

The present results profile of the moral dilemma's contrasts to some extent with results from vmPFC patients (Koenigs et al., 2007), where a specific deficit for high‐conflict dilemmas was observed. vmPFC patients only show increased utilitarian responses in moral dilemmas where a strong emotional reaction has to be overcome, and not on dilemmas where a more subtle emotional reactions has to be overcome. The authors hypothesize that vmPFC patients use compensatory strategies when judging low‐conflict moral dilemmas and do not rely on processing of social emotion cues, but rather on preserved knowledge of explicit social conventions and norms. Interestingly, these latter aspects are typically impaired in bvFTD (Bora, Walterfang, & Velakoulis, 2015; Kumfor et al., 2017).

Several anatomical subtypes of bvFTD with differential degrees of atrophy in frontal, temporal and subcortical areas have been described (Ranasinghe et al., 2016; Whitwell et al., 2009). Our sample was constituted by patients with (i) primarily frontal, (ii) primarily temporal, as well as (iii) fronto‐temporal atrophy. However, at the group level, the atrophy was concentrated around the temporal poles, but also included orbitofrontal and anterior insular regions. The samples of other studies using the same moral paradigm (Chiong et al., 2013; Koenigs et al., 2007) showed more extensive vmPFC pathology. In that sense, the present findings complement those. On the other hand, as our sample as a group mainly displayed anterior temporal atrophy, the results may not be equally applicable to the bvFTD population with more fronto‐temporal atrophy.

The imaging results are in line with recent findings in studies using a similar moral cognition paradigm. These revealed that normal subjects recruit default mode network regions during processing of personal moral dilemmas (Chiong et al., 2013; Greene et al., 2004) and that this recruitment is influenced by the salience network, in particular the anterior insula (Chiong et al., 2013). The contribution of the anterior insula has been specifically related to the processing of the emotional appraisal of moral dilemmas (Hutcherson, Montaser‐Kouhsari, Woodward, & Rangel, 2015). Furthermore, increased utilitaristic responses to personal moral dilemmas in bvFTD involves diminished recruitment of the default mode network as well as a diminished influence of the salience network on this recruitment (Chiong et al., 2013). In our study, we observed that increased utilitaristic responses in bvFTD are associated with decreased gray matter volume of areas associated with the default mode network (vmPFC). This is in line with increased utilitaristic responses to personal dilemmas in patients with ventromedial prefrontal cortex lesions (Koenigs et al., 2007). Our findings extend those results by revealing that in a neurologic sample with primarily anterior temporal structural brain pathology, it is not the locus of highest atrophy, but the integrity of the vmPFC that predicts utilitaristic behavior. Hence, our findings and those from Koenigs et al. (2007) support the notion that the vmPFC constitutes an important region in the processing of emotional characteristics of moral judgments. Furthermore, we observed that baseline activation fluctuation in the anterior insula correlates with moral deficits in bvFTD. The involvement of this region underscores the importance of emotions in moral cognition. Indeed the anterior insula has primarily been associated with awareness of feelings and internal sensations (Craig, 2009), and this area is typically atrophic in FTD (Seeley, 2010) and related to symptom severity (Van den Stock & Kumfor, 2017; Zhou & Seeley, 2014). In combination, these findings provide a structural and functional neuro‐anatomical framework for the behavioral findings that are in line with previous studies. We hypothesize that the vmPFC is structurally associated with processing personal (high‐ and low‐conflict) moral dilemmas. Preserved knowledge of explicit social and moral norms may partly compensate for damage to the vmPFC, but only when the emotional response that is triggered by the dilemma is below a critical threshold.

We hypothesize that the normal response profile in the bvFTD sample on the high‐conflict condition may reflect the level of conflict needed to activate the anterior insula sufficiently to compensate for the lower baseline activation.

On the psychological level, high‐conflict dilemmas may have the intensity to evoke emotional subjective sensations in the bvFTD group, while the intensity of the low‐conflict dilemmas was insufficient to trigger a supra‐threshold emotional experience.

In conclusion, the present study supports the threshold hypothesis of emotional deficits in bvFTD, stating that processing of subtle emotion cues is affected first. Secondly, the results provide evidence for an association between impaired face emotion recognition and increased utilitarian moral cognition in bvFTD, (suggesting a common basis for both deficits, possibly related to diminished subjective awareness of) emotional sensations. Abnormal moral cognition was neuro‐anatomically related to structural integrity of areas of the default mode network (vmPFC) and baseline activation of areas of the salience network (anterior insula). Future research should address the causal and consequential socio‐cognitive deficits, as well as more recent taxonomies of moral dilemmas (Rosas & Koenigs, 2014).

## CONFLICT OF INTEREST

None.
